# Host and geography shape microbial communities in Kenyan mosquitoes: insights from metatranscriptomics

**DOI:** 10.1128/msystems.01427-25

**Published:** 2026-01-20

**Authors:** Nailou Zhang, Evans Atoni, Raphael Nyaruaba, Paul Kibaba, Kibet Shadrack, Fei Wang, Bernard Agwanda, Zhenhua Zheng, Jun Dai, Zhiming Yuan, Han Xia

**Affiliations:** 1 Key Laboratory of Virology and Biosafety, Wuhan Institute of Virology, Chinese Academy of Sciences 74614, Wuhan, China; 2 University of Chinese Academy of Sciences 74519, Beijing, China; 3 Department of Animal Bioscience, University of Guelph 3653, Guelph, Canada; 4 Department of Medical Microbiology, Jomo Kenyatta University of Agriculture and Technology 118985, Nairobi, Kenya; 5 Mammalogy Section, National Museum of Kenya, Nairobi, Kenya; 6 State Key Laboratory of Respiratory Disease, Guangzhou Customs Technology Center 639043 , Guangzhou, China; 7 Hubei Jiangxia Laboratory, Wuhan, China; China Agricultural University Education Foundation, Beijing, China

**Keywords:** mosquito, *Aedes*, *Culex*, microbiome, virome, diversity, metagenomics, Kenya

## Abstract

Mosquitoes harbor diverse microbial communities that influence their potential to transmit pathogens. However, the ecological drivers shaping these microbiomes, particularly in under-sampled regions like Africa, remain poorly resolved. We conducted a large-scale metatranscriptomic survey of 3,940 *Aedes* and *Culex* mosquitoes from diverse ecological zones across Kenya. Our analyses revealed that viruses dominated the overall transcriptome, while bacteria exhibited the greatest taxonomic richness. Geographic location emerged as the primary driver of microbial community structure, whereas host genus identity shaped virome diversity at local or city-level scales. *Culex* mosquitoes harbored higher viral richness, particularly in coastal regions, while *Aedes* supported more diverse bacterial assemblages. Microbial co-occurrence networks exhibited distinct topologies across hosts: *Culex* networks featured cross-domain interactions and viral keystone taxa, whereas *Aedes* networks were more cohesive and robust, centered on bacterial hubs. We identified 102 distinct viruses from 24 families, including 31 putative novel RNA viruses. Segment-resolved phylogenies revealed cryptic clades within *Bunyavirales, Picornavirales,* and other lineages. Collectively, our findings highlight the scale-dependent influences of geography and host identity on mosquito microbiomes in East Africa and demonstrate the utility of metatranscriptomics in uncovering hidden microbial diversity and ecological interactions. These insights provide a foundation for ecologically informed arthropod vector surveillance and microbiome-based intervention strategies.

**IMPORTANCE:**

Mosquitoes are more than just flying syringes; they are complex ecosystems hosting a variety of microbes. Understanding what shapes this microbial world inside mosquitoes is key to developing new control strategies. Our study of nearly 4,000 mosquitoes from Kenya reveals that where a mosquito lives matters most for its overall microbial makeup, but its genus dictates which viruses it carries. We discovered that different mosquito types have distinct microbial social networks: one type has a fragile network centered on viruses, while the other has a resilient network built around bacteria. This means that strategies to disrupt disease transmission by targeting mosquito microbes may need to be tailored to a specific mosquito genus. Our work provides a map of these microbial ecosystems, highlighting potential new viruses and offering insights for future public health surveillance and interventions.

## INTRODUCTION

Vector-borne diseases such as malaria, dengue, Zika, chikungunya, yellow fever, West Nile fever, Japanese encephalitis, and lymphatic filariasis collectively cause over 700,000 deaths annually, with mosquitoes serving as major arthropod vectors of numerous medically important pathogens (1). Intensifying urbanization, global climate change, and the increasing potential for long-distance dispersal of mosquitoes via atmospheric currents are contributing to the expansion of mosquito habitats and the geographical spread of associated diseases (2–4). Thus, continued surveillance and comprehensive profiling of mosquito-associated microbiomes are essential to identify potential pathogenic threats and elucidate the ecological drivers of pathogen maintenance and transmission.

Endogenous microbial communities in mosquitoes can profoundly affect the host vectorial capacity, immunity, and competence for pathogen transmission (5–7). Among these, *Wolbachia* spp. have attracted particular attention due to their ability to inhibit the replication and transmission of several arboviruses, including dengue, chikungunya, and Zika viruses in *Aedes,* through mechanisms such as competitive exclusion and immune priming (8–10). Consequently, *Wolbachia*-based biocontrol strategies have been implemented in multiple regions and have demonstrated substantial reductions in arboviral incidence in target populations (11–13). Beyond *Wolbachia*, other members of the mosquito microbiota, including midgut-associated bacteria, insect-specific viruses (ISVs), and eukaryotic symbionts, such as microsporidia, have also been implicated in modulating host–pathogen dynamics (14–21). Nevertheless, the overall diversity, structure, and functional roles of these microbial communities and their inter-species interactions remain incompletely characterized, particularly in wild mosquito populations.

Recent advances in metatranscriptomics have enabled unbiased, high-resolution profiling of active microbial constituents spanning bacteria, viruses, fungi, and protists within individual mosquitoes, while simultaneously capturing host–microbe and microbe–microbe interactions *in situ* (22–27). High-throughput sequencing efforts have considerably expanded our knowledge of mosquito-associated viromes across global settings (23, 25, 28–33). However, much of this work has focused narrowly on virus discovery, with limited attention to the broader microbial ecosystem or to the ecological drivers that shape microbiome composition and function. Crucial variables, such as host genus identity, local habitat conditions, seasonal dynamics, and host sex, are seldom incorporated into such analyses, thereby limiting our capacity to anticipate patterns of pathogen emergence and transmission.

By integrating metatranscriptomic data from 3,940 *Aedes* and *Culex* mosquitoes across Kenya, we aimed to (i) characterize active microbial communities, (ii) quantify the influence of geography, time, and host genus, (iii) compare cross-domain microbial networks, and (iv) assess the diversity and relevance of RNA viruses. Our findings delineate the ecological drivers of mosquito microbiomes, with implications for vector surveillance and pathogen interventions.

## RESULTS

### Sampling overview and microbiome composition

**Fig 1:**
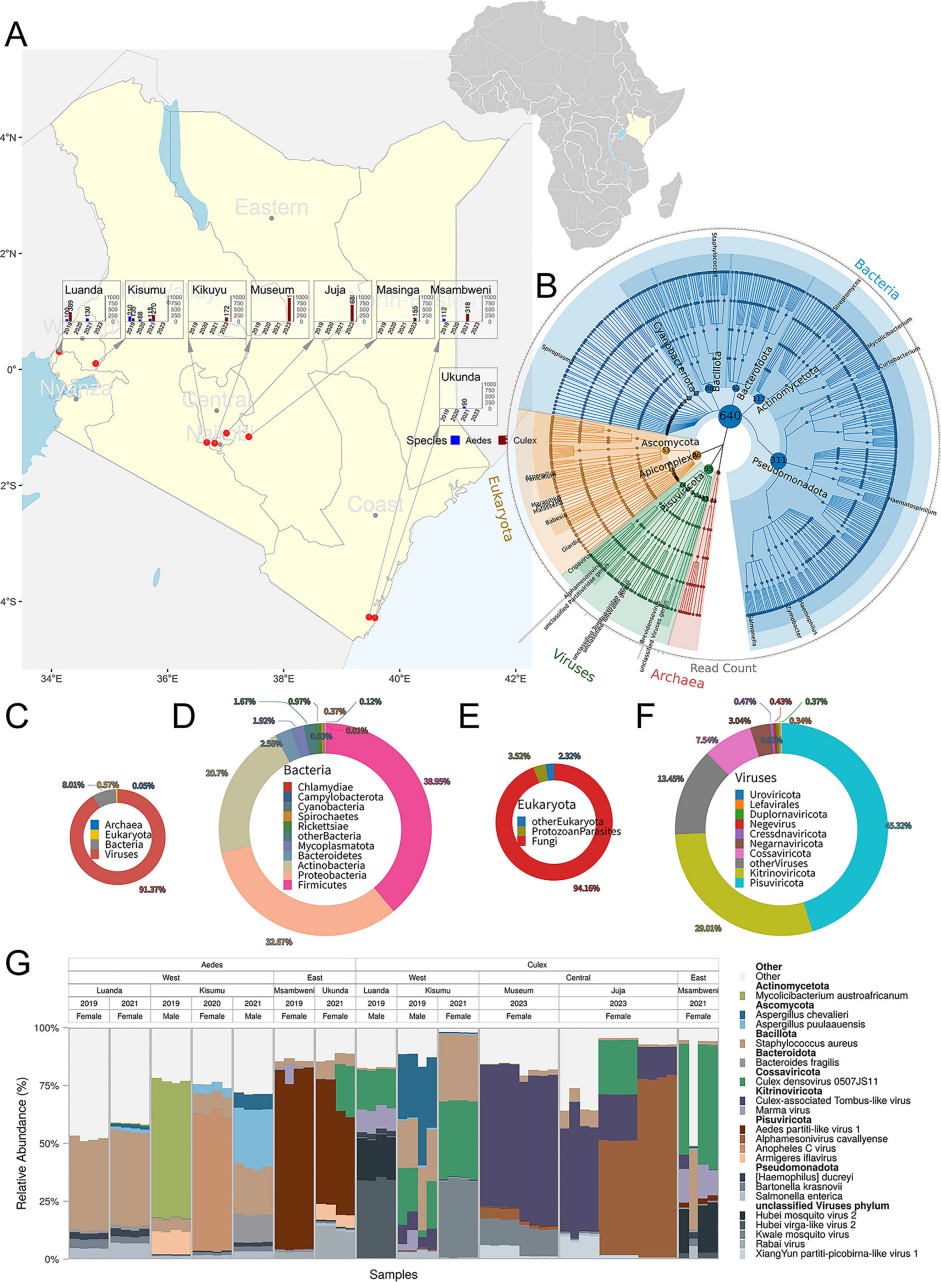
Taxonomic overview of the mosquito-associated microbiome across Kenya. (**A**) Sampling distribution of *Aedes* and *Culex* mosquitoes across eight Kenyan locations from 2019 to 2023. Insets show genus and year-specific sampling per site. (**B**) Integrated microbial composition, including bacteria, eukaryotic microbes, viruses, and archaea across all mosquito samples. The central circle shows species counts per phylum; outer labels mark the most abundant species per phylum; the outermost ring presents aggregated relative abundance. (**C**) Proportion of sequencing reads assigned to each microbial domain across all samples. (**D–F**) Relative abundance of detected taxa at the phylum level within bacterial (**D**), eukaryotic (**E**), and viral (**F**) domains. (**G**) Relative abundance profiles of the top 20 microbial taxa across individual mosquito samples. See Table S2 for the full abundance matrix of microbial taxa.

Between 2019 and 2023, a total of 3,940 adult mosquitoes (genera *Aedes* and *Culex*) were collected from eight ecologically diverse urban and peri-urban sites across Kenya, including Luanda, Kisumu, Kikuyu, Museum, Juja, Masinga, Msambweni, and Ukunda. This broad spatiotemporal sampling framework facilitated an integrated analysis of host- and geography-associated microbial patterns across various ecological settings (Fig. 1A). Mosquitoes were deliberately pooled based on host genus, sex, sampling time, and collection site to create 21 distinct biological pools. These pools were substantial, ranging from 88 to 600 individual mosquitoes each (Table S1). Critically, to ensure data reliability and address potential technical variation, each of the 21 biological pools was split into four technical replicates for library preparation and sequencing, totaling 84 sequencing libraries.

Metatranscriptomic sequencing of whole mosquitoes revealed that Kenyan mosquito populations harbor a highly diverse microbiota spanning all four microbial domains (Bacteria, Archaea, Eukaryota, and Viruses). Taxonomically, bacterial taxa contributed the highest number of distinct species, followed by eukaryotic microbes, viruses, and archaeal lineages. At the phylum level, bacterial communities were predominantly composed of *Pseudomonadota* (formerly *Proteobacteria*) and *Actinomycetota*, with *Bacillota* (*Firmicutes*) also contributing substantially. Among eukaryotic microbes, *Ascomycota*, a major fungal phylum, was the most represented, while a minor but notable fraction included protozoan lineages, such as *Apicomplexa*, *Euglenozoa*, *Amoebozoa*, and *Ciliophora* (Fig. 1B).

In terms of relative sequence abundance, viral reads dominated the metatranscriptomes, accounting for approximately 91.37% of total non-host reads, followed by bacterial (8.01%), eukaryotic (0.57%), and archaeal reads (0.05%) (Fig. 1C). The virome was largely composed of RNA viruses belonging to the phyla *Pisuviricota* (45.32%) and *Kitrinoviricota* (29.01%), with additional contributions from single-stranded DNA viruses classified under *Cossaviricota* (Fig. 1F). Within the bacterial compartment, *Bacillota* (*Firmicutes,* 38.95%), *Pseudomonadota* (32.67%), and *Actinomycetota* (20.7%) were the most abundant phyla, reflecting the coexistence of both Gram-positive and Gram-negative taxa across host and location gradients (Fig. 1D). Fungal communities were dominated by *Ascomycota* (94.16%), whereas protozoan groups comprised a small but diverse minority (Fig. 1E).

At the species level, *Staphylococcus aureus* and *Staphylococcus pseudintermedius*, both Gram-positive opportunistic pathogens, were frequently detected and widely distributed across mosquito populations. Viral communities were similarly structured by dominant species, with a *Culex*-associated tombus-like virus found at high prevalence in *Culex* samples across nearly all sites, suggesting both strong host specificity and wide spatial dissemination (Fig. 1G).

### Drivers of bacterial diversity and composition

**Fig 2:**
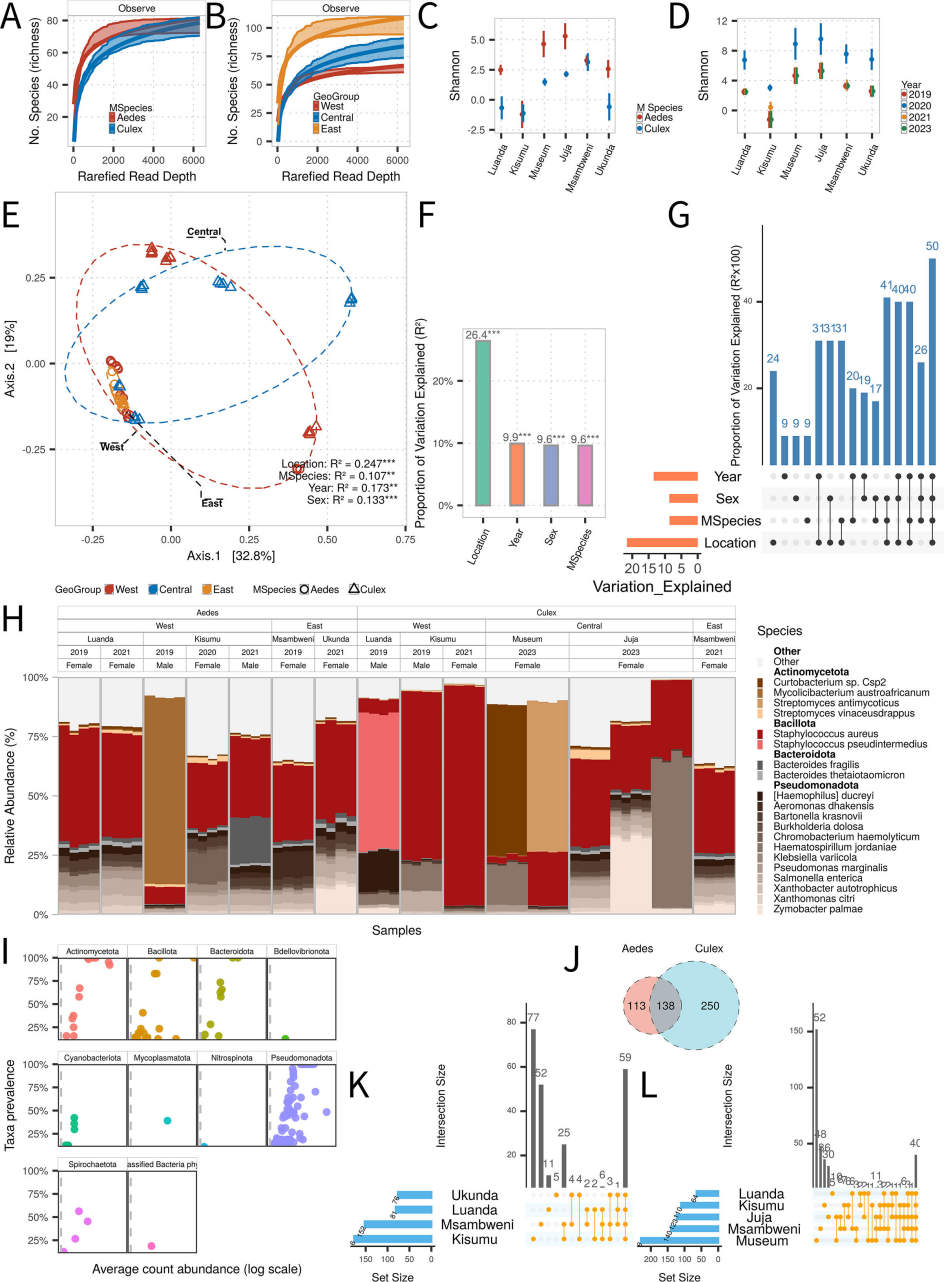
Ecological drivers of bacterial diversity and community composition in Kenyan mosquitoes. (**A, B**) Rarefaction curves showing bacterial species richness across mosquito genus (**A**) and sampling locations (**B**). (**C, D**) Predicted Shannon diversity index values for bacteria across mosquito genus (**C**) and years (**D**), based on stepwise AIC modeling. (**E**) Principal coordinates analysis (PCoA) of bacterial beta diversity based on Bray-Curtis distances; PERMANOVA *R*^2^ and *P* values are shown for each grouping variable. Significance: **P < 0.001, *P < 0.01, *P* < 0.05. (**F**) Distance-based redundancy analysis (db-RDA) illustrating the proportion of variance in bacterial composition explained by geography, year, sex, and mosquito genus. (**G**) Variation partitioning (varpart) and Venn diagram showing shared and unique contributions of each factor. (**H**) Relative abundance of the top 20 bacterial species across all mosquito samples. (**I**) Prevalence versus mean relative abundance plot for bacterial phyla across all samples. (**J–L**) Venn diagrams showing species-level bacterial overlap between *Aedes* and *Culex* (**J**), among *Aedes* across locations (**K**), and among *Culex* across locations (**L**).

To explore the ecological factors underlying bacterial diversity in Kenyan mosquitoes, we assessed alpha diversity using rarefaction curves and the Shannon index. Both host genus and geographic origin significantly influenced bacterial richness. *Aedes* consistently exhibited higher alpha diversity than *Culex* across most locations (Fig. 2A and B; Fig. S1A). Stratified analysis further revealed that *Aedes* populations from central Kenya (Museum and Juja) harbored particularly richer bacterial communities (Fig. 2C). Temporal analysis also showed that mosquitoes sampled in 2020 exhibited greater bacterial diversity compared to other years, suggesting that interannual environmental fluctuations influence microbial colonization (Fig. 2D).

We conducted beta-diversity analysis using Bray-Curtis dissimilarity and PERMANOVA to assess compositional differences. Geographic location explained the largest proportion of variance (24.7%), followed by sampling year (17.3%), mosquito sex (13.3%), and host genus identity (10.7%) (Fig. 2E). These findings were corroborated by distance-based redundancy analysis (db-RDA), which yielded comparable explanatory values (geography: 26.4%; sampling year: 9.9%; mosquito sex: 9.6%; host genus: 9.6%) (Fig. 2F). Variation partitioning (varpart) analysis revealed that these four variables collectively accounted for approximately 50% of the variation in bacterial composition. This suggests that the interplay between environmental and host-related variables contributes jointly to shaping mosquito-associated bacterial communities (Fig. 2G). The remaining unexplained variance implies the potential influence of additional, unmeasured ecological factors.

Core bacterial phyla, including *Actinomycetota*, *Bacillota*, *Bacteroidota*, and *Pseudomonadota,* were consistently prevalent across all regions, reflecting a stable background of mosquito-associated taxa (Fig. 2I). Within these phyla, several species displayed high relative abundance and broad geographic distribution. The Gram-positive species *Staphylococcus aureus* and *Staphylococcus pseudintermedius* were frequently detected in both *Aedes* and *Culex* samples, and across nearly all regions, confirming their role as core members of the mosquito microbiota. These findings were consistent with global abundance profiles across the data set (Fig. 1G). In addition, members of the phylum *Pseudomonadota* also contributed substantially to bacterial load in most samples (Fig. 2H and I).

At the species level, 138 bacterial species were shared between *Aedes* and *Culex*, while many others were genus-exclusive (Fig. 2J). Analysis of species overlap across sampling locations revealed that the majority of bacterial taxa exhibited strong geographic specificity, consistent with the role of regional environmental conditions. *Aedes* shared 59 species among different regions, slightly more than the 40 species shared among *Culex* (Fig. 2K and L, respectively).

### Drivers of viral diversity and composition

**Fig 3:**
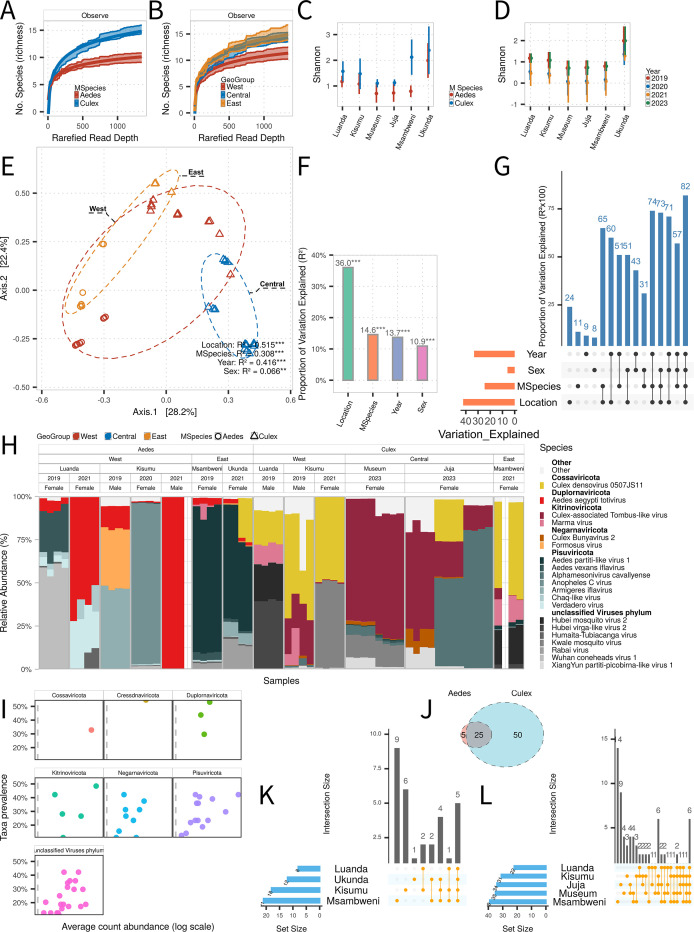
Ecological drivers of viral diversity and community composition in Kenyan mosquitoes. (**A, B**) Rarefaction curves for viral species richness across mosquito genus (**A**) and locations (**B**). (**C, D**) Predicted Shannon diversity values for viral communities across mosquito genus (**C**) and years (**D**). (**E**) PCoA based on Bray-Curtis distances of viral composition, with PERMANOVA results annotated. (**F**) db-RDA showing the relative contribution of geographic region, year, host genus, and sex to viral community structure. (**G**) Variation partitioning and Venn diagram illustrating individual and shared effects on viral composition. (**H**) Relative abundance of the top 20 viral species across samples. (**I**) Prevalence and mean abundance plot for viral phyla across samples. (**J–L**) Venn diagrams of viral species shared between the mosquito genus (**J**), among *Aedes* across locations (**K**), and among *Culex* across locations (**L**).

To identify factors influencing virome diversity, we performed rarefaction and Shannon diversity analyses. In contrast to the bacterial communities, *Culex* harbored significantly greater viral species richness than *Aedes* across sampling sites (Fig. 3A and C; Fig. S1C). Although geographic origin was dominant overall, *Culex* populations from coastal areas (Msambweni and Ukunda) exhibited especially high viral diversity (Fig. 3C; Fig. S1D). This suggests that localized ecological factors may shape virome richness more strongly than broader regional classifications, such as western, central, or eastern Kenya (Fig. 3B; Fig. S1C). Mosquitoes collected in 2019 and 2023 exhibited higher virome diversity compared to those sampled in 2020 and 2021 (Fig. 3D). This temporal fluctuation may be partially attributed to the large-scale public health interventions implemented during the COVID-19 pandemic.

To quantify the relative contribution of environmental and host-associated factors, we assessed beta diversity using Bray-Curtis dissimilarity, followed by PERMANOVA. The results indicated that geography explained the largest portion of variance in viral composition (51.5%), followed by sampling year (41.6%), mosquito genus (30.8%), and mosquito sex, which contributed only marginally (0.07%) (Fig. 3E). These trends were supported by db-RDA, which showed that geography accounted for 36% of variation, sampling year for 14.6%, mosquito genus for 13.7%, and sex for 10.9% (Fig. 3F). Combined, these four variables explained approximately 82% of the variation in viral community structure (Fig. 3G), suggesting that virome composition is strongly shaped by a complex interplay of spatial, temporal, and host identity factors.

Most viruses were detected at relatively low prevalence (less than 50% of samples for any given virus) (Fig. 3I). However, their distribution patterns were non-random. Many viral taxa displayed highly localized distributions and often dominated in only a single mosquito genus or site (Fig. 3H). At the species level, approximately 83% of viruses found in *Aedes* were also detected in *Culex*, suggesting considerable sharing. Conversely, nearly two-thirds of the viruses identified in Culex appeared to be genus-specific, indicating that *Culex* mosquitoes harbor a broader and more unique viral repertoire (Fig. 3J).

Set-based Venn analyses revealed further geographic structuring within mosquito genera. In *Aedes*, several viruses, including *Aedes vexans iflavirus, Aedes aegypti totivirus*, *Verdadero virus*, *Chaq-like virus*, and *Humaita-Tubiacanga virus,* were widely distributed across multiple locations, indicating a potential core virome in this genus (Fig. 3K). In *Culex*, a different set of viruses dominated across sites, such as *XiangYun nudi-like virus*, *Point-Douro narna-like virus*, *Alphamesonivirus cavallyense*, *Guadeloupe Culex rhabdovirus*, *XiangYun circovirus-like virus*, and *Guadeloupe Culex tymo-like virus*, suggesting a host-genus-specific and spatially structured viral community (Fig. 3L).

### Inter-microbial network structure and keystone taxa

**Fig 4:**
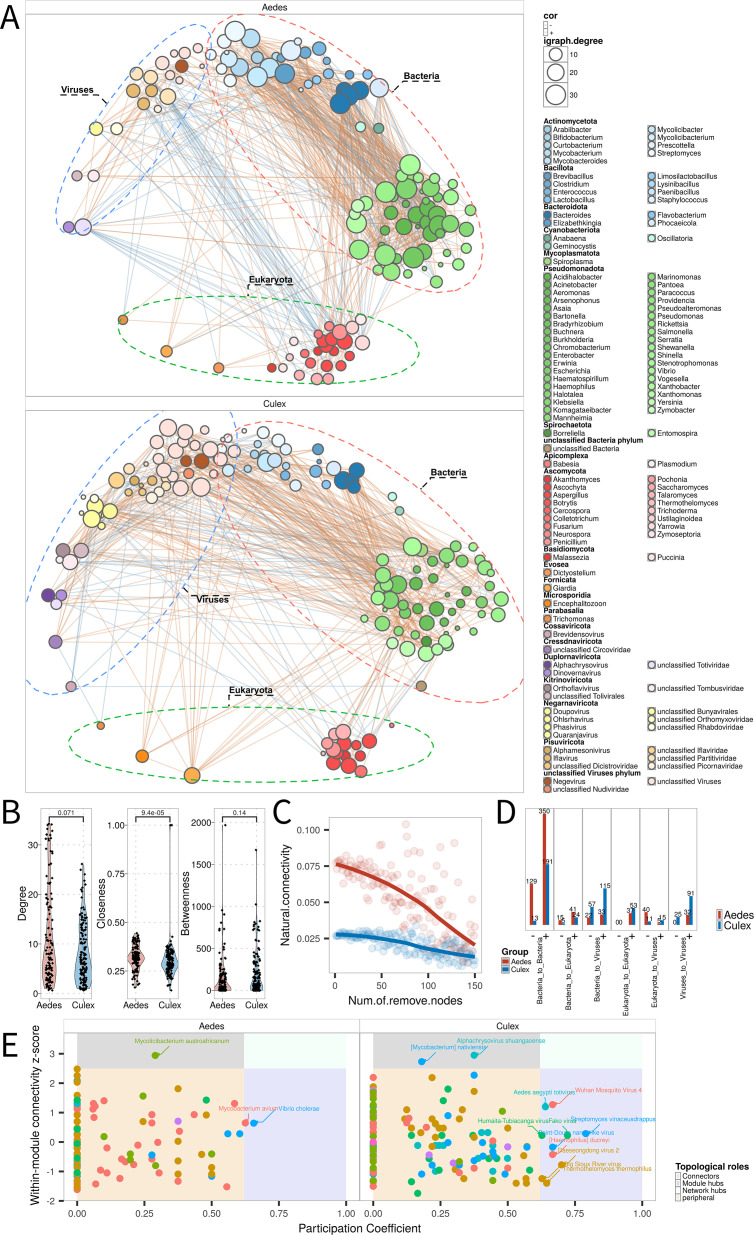
Microbial co-occurrence networks in *Aedes* and *Culex* mosquitoes. (**A**) Co-occurrence networks constructed using SparCC based on bacteria, viruses, and eukaryotic microbes in *Aedes* and *Culex*. Node color represents taxonomic phylum; edge color indicates correlation sign (orange: positive, blue: negative); node size is proportional to node degree. (**B**) Comparative distribution of network centrality metrics—degree, closeness, and betweenness—for *Aedes* and *Culex*. (**C**) Simulated node deletion (robustness analysis) assessing network fragmentation under stepwise removal of central nodes. (**D**) Proportions of positive and negative correlations among microbial domains within *Aedes* and *Culex* networks. (**E**) ZiPi (Zipf-Pi) analysis identifying the topological roles of microbial taxa in each network. Colors denote role categories (peripheral, connector, module hub, network hub) based on within- and among-module connectivity.

To explore the ecological architecture of mosquito microbial communities, we constructed co-occurrence networks based on non-archaeal taxa for *Culex* and *Aedes* populations separately. Utilizing SparCC-inferred correlation matrices and circular network visualizations (Fig. 4A), we observed striking differences between the two genera. *Aedes* networks were characterized by higher intra-bacterial connectivity, reflecting a densely interconnected bacterial community. Conversely, *Culex* networks displayed a greater number of cross-domain associations, particularly between bacteria and viruses, as well as among viruses themselves (Fig. 4A).

To quantify network topology, we calculated three centrality metrics: degree (indicating the number of connections per node), closeness (reflecting how quickly information can spread), and betweenness (capturing a node’s role in connecting subnetworks). All three metrics were significantly elevated in *Aedes* networks, indicating a more cohesive and integrated microbial structure (Fig. 4B). Targeted node-deletion simulations, conducted to assess robustness, showed that *Aedes* networks exhibited higher structural resilience, maintaining modular integrity despite the removal of highly connected taxa. In contrast, *Culex* networks were more susceptible to fragmentation, suggesting a reliance on a smaller number of critical nodes (Fig. 4C).

Analysis of correlation polarity further underscored divergent ecological strategies. In *Aedes*, the majority of statistically significant positive correlations occurred among bacterial taxa, suggesting predominantly cooperative or co-stabilizing bacterial interactions. On the other hand, *Culex* networks were marked by complex patterns of both positive and negative correlations that spanned bacteria–virus and virus–virus interactions (Fig. 4D).

To identify keystone taxa that maintain network integrity, we employed Zipf-Pi (ZiPi) analysis, which classifies nodes based on their topological roles, such as module hubs, connectors, and peripherals. In *Aedes*, bacterial taxa dominated the central roles: *Mycolicibacterium austroafricanum* served as a module hub, while *Mycobacterium avium* and *Vibrio cholerae* functioned as global connectors linking distinct microbial sub-communities. These results reinforce the importance of bacterial taxa in shaping and stabilizing the *Aedes* microbiome (Fig. 4E). In contrast, the *Culex* network architecture depended on a combination of bacterial and viral taxa. Several ISVs, including *Wuhan mosquito virus 4*, *Fako virus*, and *Daeseongdong virus*, were identified as topologically central nodes, suggesting that viral taxa play integral roles in organizing *Culex* microbial communities (Fig. 4E).

### Mosquito virome

A comprehensive analysis of the metatranscriptomic data sets revealed 102 distinct viral species in Kenyan mosquito populations, spanning 24 viral families. RNA viruses were assigned to 17 families, including well-characterized insect-specific lineages, such as *Spinareoviridae*, *Orthomyxoviridae*, *Picornaviridae*, and *Flaviviridae*. DNA viruses were detected from five families, including *Circoviridae* and *Parvoviridae*.

**Fig 5:**
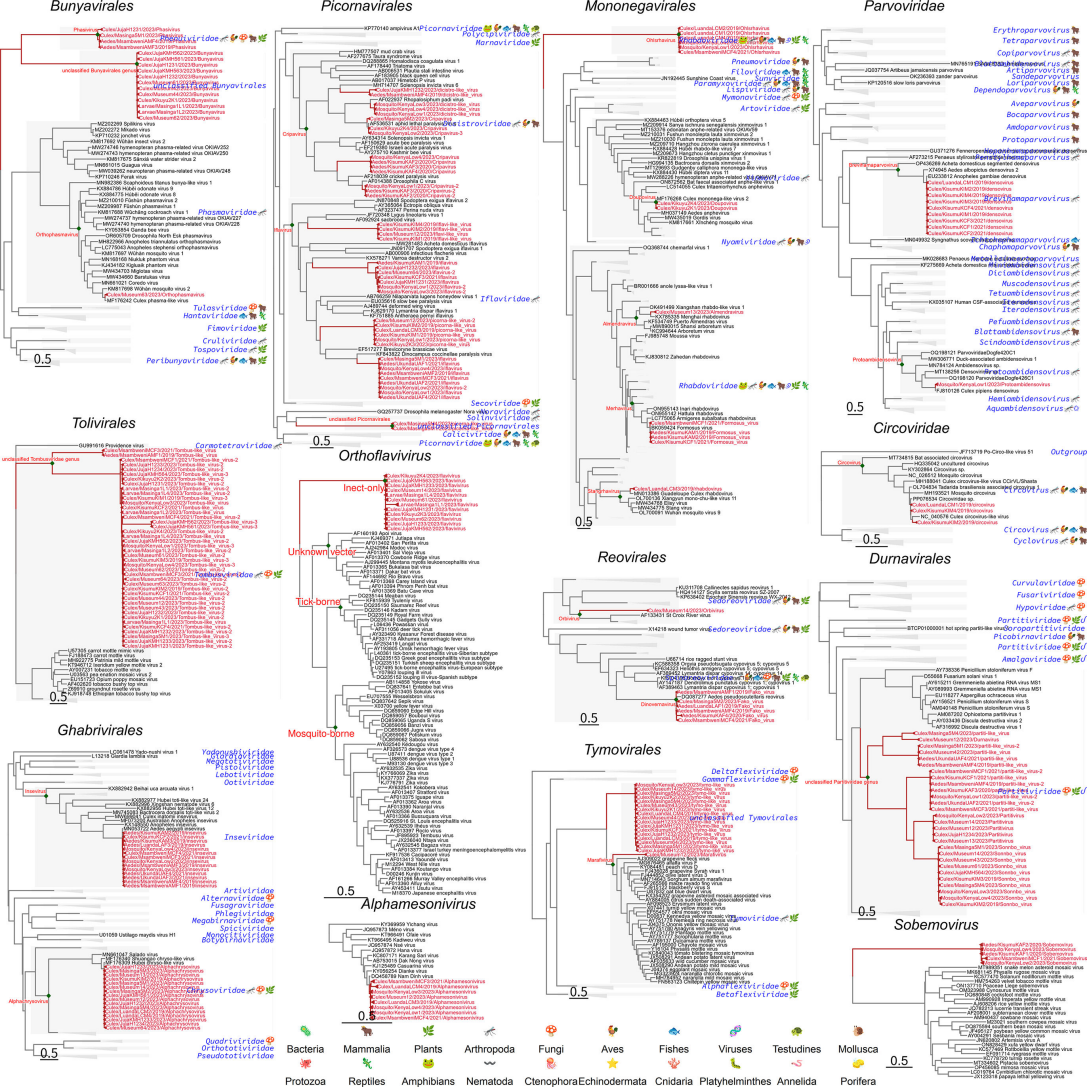
Phylogenetic diversity of RNA and DNA viruses identified from Kenyan mosquitoes. Maximum likelihood phylogenetic trees were reconstructed for representative viral families detected in this study. Red labels indicate virus sequences identified from Kenyan mosquitoes, annotated by host genus/location/year/closest reference strain. Green nodes indicate genus-level clades; blue icons represent host taxonomy of referenced viral lineages. Collapsed triangles denote compressed clades for families or genera not represented by Kenyan sequences.

Importantly, 31 viral species (nearly one-third of the detected virome) shared less than 80% nucleotide identity with any previously described reference genomes, indicating they represent novel or highly divergent viral lineages. None of these viruses is currently associated with human or vertebrate pathogenicity (Fig. 5). Phylogenetic reconstruction of representative viral proteins (e.g., RNA-dependent RNA polymerase and capsid proteins) confirmed that all sequences clustered within insect-associated viral clades. However, several clades exhibited substantial evolutionary divergence. Within *Bunyavirales*, we identified a lineage closely related to—but phylogenetically distinct from—the family *Phenuiviridae*, potentially representing a novel family- or genus-level group. Similarly, within *Picornavirales*, two previously unrecognized clades were identified that were most closely related to *Solemoviridae* and unclassified *Nora*-like viruses. These findings suggest that the current taxonomy of these viral orders may underestimate their true diversity and require further revision.

Particularly high intra-family diversity was observed in several RNA virus families. Members of *Iflaviridae* were distributed across four phylogenetically distinct clades, suggesting the presence of multiple deeply diverging lineages within mosquitoes in this region. In addition, viruses assigned to the genus *Cripavirus* (family *Dicistroviridae*) were grouped into five separate clades, highlighting the extensive hidden diversity and likely undersampled phylogenetic space within these taxa.

### Segment-based phylogenetics of five segmented viruses

**Fig 6:**
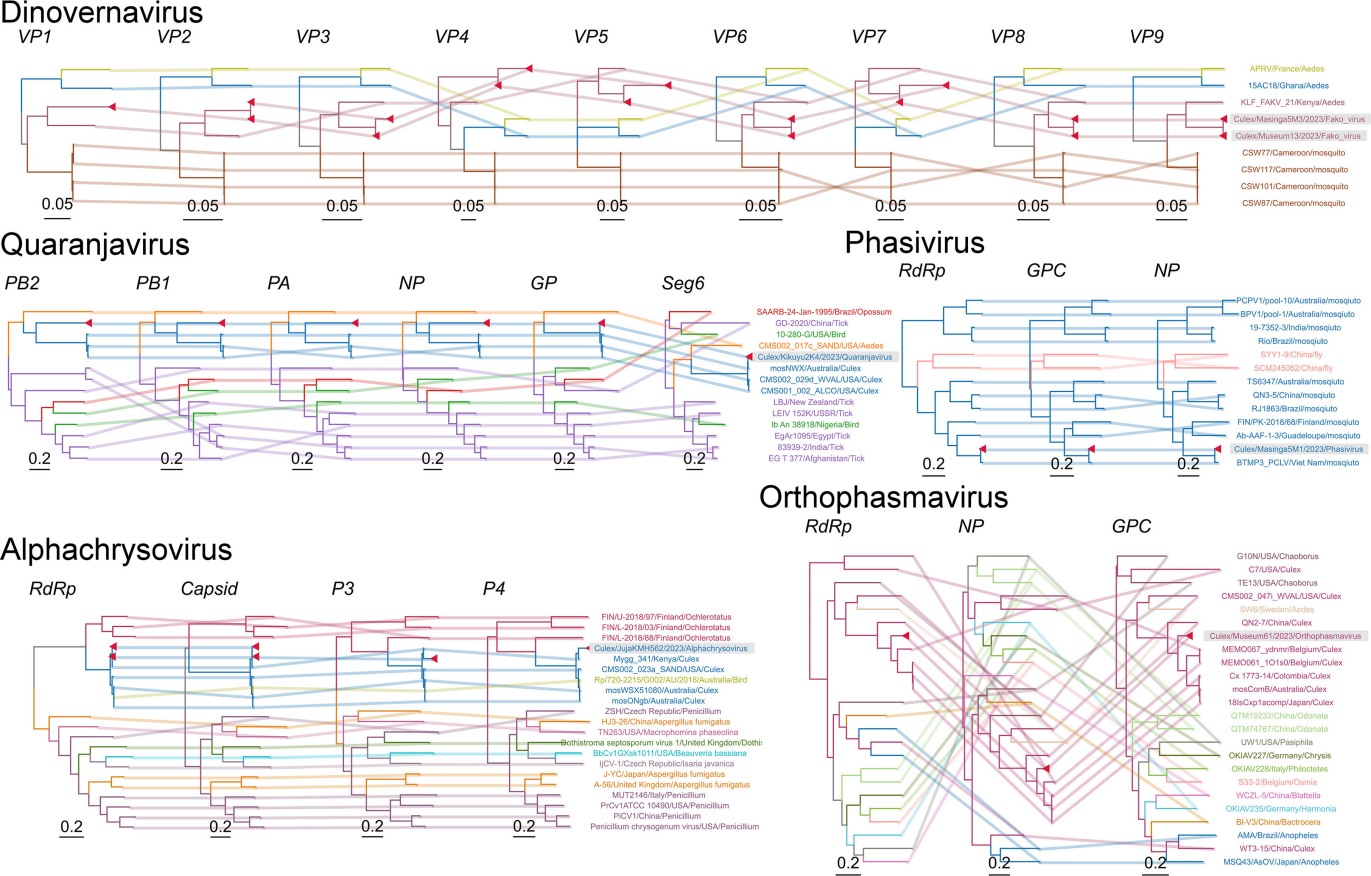
Segment-based phylogenies of segmented RNA viruses detected in Kenyan mosquitoes. Phylogenetic trees of genome segments for five segmented viruses: Quaranjavirus, Dinovernavirus, Alphachrysovirus, Phasivirus, and Orthophasmavirus. Colored lines connect genome segments from the same virus isolate; node and label colors reflect mosquito genus. Red triangles and a gray background highlight virus sequences detected in this study.

To resolve the evolutionary relationships of segmented RNA viruses detected in Kenyan mosquitoes, we performed segment-resolved phylogenetic analyses on five families or genera of segmented viruses, including *Quaranjavirus*, *Dinovernavirus*, *Alphachrysovirus*, *Phasivirus*, and *Orthophasmavirus* (Fig. 6).

*Quaranjaviruses* (family *Orthomyxoviridae*) possess eight canonical segments. In our data set, six segments were consistently recovered from multiple *Culex* pools, including PB2, PB1, PA, NP, GP, and segment 6. Segment 3 was only recovered in a single sample, possibly due to low expression or structural divergence. Phylogenetic reconstruction based on the PB1 and NP segments revealed a clear separation between mosquito-derived and tick-associated *Quaranjaviruses*. Within mosquito-borne clades, viral sequences clustered according to host genus, with *Culex*- and *Aedes*-associated viruses forming distinct sister lineages. This observation suggests either parallel host adaptation or ancient divergence, followed by host-specific maintenance.

*Dinovernaviruses* are composed of nine double-stranded RNA segments, which were exclusively detected in *Culex* mosquitoes from this study. The assembled segments showed greater than 99% nucleotide identity to Fako virus, a known mosquito-associated reovirus. Phylogenetic trees based on major capsid and RNA polymerase segments showed grouping by both host and sampling location, supporting a pattern of local persistence and host specificity.

*Alphachrysoviruses*, typically composed of three or four dsRNA segments, were identified in *Culex* samples from Juja. These sequences clustered closely with strains previously isolated from *Aedes* mosquitoes in the United States of America and Australia. Notably, related *Alphachrysoviruses* have also been reported from avian guano and hemipteran insects, suggesting that members of this viral group may function as persistent symbionts across diverse insect taxa. The apparent cross-order presence raises the possibility of ecological transmission via shared habitats or food-web linkages rather than strict host specificity.

*Phasiviruses* (family *Phenuiviridae*) contain three negative-sense RNA segments. Kenyan *Culex*-derived Phasivirus sequences grouped with isolates from geographically distant locations, including India, Vietnam, Guadeloupe, and South America.

*Orthophasmaviruses* (family *Phasmaviridae*), also with three segments, were recovered from *Culex* mosquitoes and showed phylogenetic affinities with previously reported strains from Australia, Belgium, Japan, and Colombia. The consistent association with *Culex* mosquitoes across continents suggests that this viral lineage is both geographically widespread and host-associated.

### Host and geographic associations of mosquito viruses

**Fig 7:**
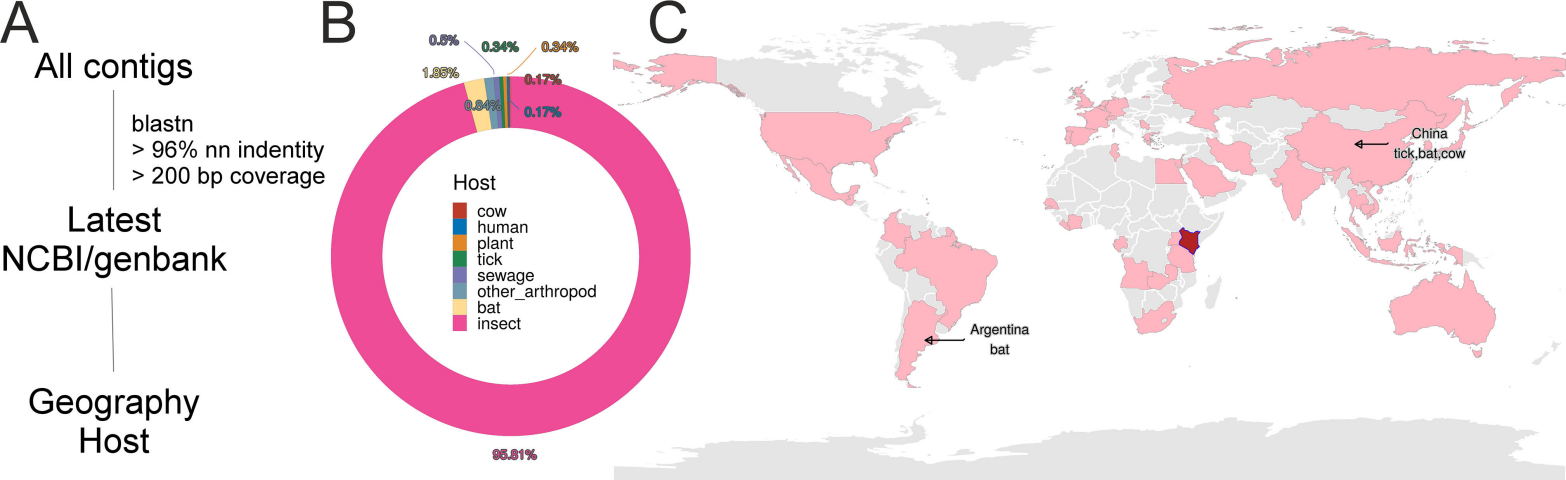
Geographic and host distribution of closely related viral sequences. (**A**) Workflow for identifying nearest NCBI reference sequences to contigs assembled from Kenyan mosquito viruses. (**B**) Proportion of reference sequences by host organism based on top BLAST hits (≥96% identity, ≥200 bp coverage). (**C**) Global map showing the geographic origin of the best BLAST matches, suggesting potential ecological overlap or cross-region connectivity.

To explore the potential host associations and global distribution of viruses identified in Kenyan mosquitoes, we performed taxonomic assignment of all assembled viral contigs through BLASTn alignment against the NCBI nucleotide (nt) database (Fig. 7A). Only high-confidence matches were retained (defined as ≥96% nucleotide identity over at least 200 base pairs). Among the resulting annotations, 95.8% of matched sequences originated from insects, and over 99% of those were specifically derived from mosquito-associated data sets, strongly supporting the insect-specific nature of the virome described here (Fig. 7B). A small proportion of viral hits (1.85%) were associated with non-insect vertebrate hosts, including bats and cattle.

Geospatial analysis of the best-matching reference sequences revealed that viruses detected in this study are broadly distributed across multiple continents (Fig. 7C). In particular, *Cripavirus* and *Dicistrovirus* sequences, typically considered insect-specific, were found to have high similarity to viral strains previously isolated from bat or cow fecal or oral swab samples in China and Argentina. Although cross-domain transmission is unlikely given the evolutionary constraints of these viruses, such observations raise the possibility of indirect ecological associations or sampling contamination in source data sets.

## DISCUSSION

In Kenyan mosquitoes, we found that viruses dominate microbial abundance, while bacteria exhibit the greatest richness. Geography primarily structured communities nationally, but the mosquito genus dictated local viral composition, and temporal variation shaped bacteria. Our results underscore a multi-scale ecological framework for microbiome assembly in vectors.

### Scale-dependent drivers of microbiome structure: geography vs host identity

Our findings demonstrate a clear scale-dependent pattern in the structuring of mosquito-associated microbial communities: geographic location emerged as the primary determinant at broad spatial scales, while host genus identity exerted a greater influence on local or city-level scales. For instance, *Culex* mosquitoes in Kenya harbored significantly higher viral richness than *Aedes*, echoing the findings of Pan et al. (31), who reported that both host phylogeny and geography shaped virome diversity along a ~4,000 km transect across mainland China (31). Their study also documented substantial viral sharing across mosquito genera and geographic hotspots, emphasizing the importance of long-range mosquito dispersal in shaping virome patterns.

Conversely, studies conducted in ecologically homogeneous or spatially restricted settings have reported different patterns. Shi et al. (34) found that *Aedes e*xhibited greater viral diversity than *Culex* in Guadeloupe (34), and similar trends were observed in Hainan Island and Yunnan Province, China (25, 29). Liu et al. (29) particularly highlighted the importance of hierarchical spatial sampling and cautioned against the pooling of samples across geographic regions or host genus, as this practice may obscure fine-scale ecological signals (29).

Our results reinforce the concept of spatial scale dependence in mosquito microbiome research. In large, topographically and climatically diverse regions—such as Kenya—geographic variation becomes the dominant structuring force. However, in ecologically stable or confined systems, host genus identity often supersedes geography in shaping microbial community composition. This scale-specific dynamic underscores the necessity for stratified sampling designs that incorporate environmental variables, such as climate, elevation, hydrological connectivity, land use, and mosquito dispersal capacity. Only by integrating these factors can we accurately resolve the ecological processes driving mosquito microbiome and virome structure and ultimately improve the design of vector surveillance and control programs.

### Microbial interaction networks as a tool for identifying intervention targets

Microbial co-occurrence networks offer a powerful framework for understanding ecological interactions—such as cooperation, competition, and predation-like dynamics—within mosquito-associated microbiomes (35). In this study, we constructed microbial interaction networks for *Culex* and *Aedes* mosquitoes and observed distinct patterns that suggest divergent ecological architectures and intervention strategies for these two genera. Our analyses revealed that bacteria–bacteria interactions dominated the microbial networks of both mosquito groups. However, *Culex* networks featured a higher proportion of bacteria–virus and virus–virus associations, in line with prior findings that ISVs can integrate into host microbiomes and contribute to stability of co-infection (16). Notably, we identified several keystone viral taxa in *Culex*, including *Wuhan Mosquito Virus 4*, *Fako virus*, and *Daeseongdong virus*, which exhibited high topological centrality. Simulated node removal experiments demonstrated that the elimination of these viral nodes substantially disrupted network modularity, highlighting their importance as structural hubs and potential microbial control targets.

In contrast, *Aedes* networks were characterized by greater overall structural resilience to node perturbation and appeared to rely more heavily on bacterial nodes to maintain network integrity. This suggests that while *Culex* networks may be more vulnerable to targeted disruption of viral components, *Aedes* virome stability is more tightly coupled with bacterial community dynamics. Such differences imply that microbiome-based intervention strategies should be tailored to mosquito genus: in *Culex,* targeting viral hubs may effectively destabilize the network, whereas in *Aedes*, modulation of bacterial taxa may provide a more viable route for interfering with virus persistence or transmission.

### Conclusion

Our findings reveal a spatially and taxonomically structured mosquito microbiome across ecologically diverse regions of Kenya. By uncovering scale-dependent roles of geography and host identity, we provide a framework for geographically tailored microbial surveillance. The integration of metatranscriptomics with microbial network analysis offers a novel strategy for identifying microbial biomarkers and potential intervention targets. Future efforts should aim to experimentally validate key virome components, incorporate ecological metadata, and explore microbiome engineering approaches to reduce spillover risk from poorly characterized viruses.

## MATERIALS AND METHODS

### Mosquito sampling and sample processing

Mosquitoes were collected from six cities across Kenya using UV light traps (Fig. 1A). Individuals showing visible signs of blood feeding were excluded. Initial classification into *Aedes* and *Culex* was performed morphologically. Mosquitoes were flash-frozen in liquid nitrogen and stored at −80°C.

To ensure robust metatranscriptomic profiling, mosquitoes were pooled based on host genus, sex, sampling time, and collection site. The total number of mosquitoes processed and the composition of each pool are detailed in Table S1. Molecular verification was performed to confirm the genetic purity of the pools. Assembled host contigs (>200 bp) were analyzed for the cytochrome c oxidase subunit I (*COI*) gene (Pfam domain: PF000115, identified via HMMscan and BLASTn). This analysis confirmed the absence of inter-generic cross-contamination between *Aedes* and *Culex* pools.

### RNA extraction, library construction, and sequencing

Total RNA was extracted using the RNeasy Mini Kit (Qiagen). Libraries were prepared using the Illumina TruSeq Stranded Total RNA Kit (Illumina Inc.), utilizing random hexamer primers for total RNA profiling. Each of the 21 biological pools was split into four technical replicates for library preparation, generating a total of 84 indexed libraries. Libraries were pooled, normalized to 4 nM, and sequenced using 1.5 pM on the Illumina NextSeq 550 platform (paired-end sequencing, BIO-Gene Seq Kenya).

### Metatranscriptomic processing, assembly, and taxonomic profiling

Raw reads were processed using fastp (v0.23.2) for adapter trimming, quality, and length filtering. Host-derived sequences were subtracted by classification against the custom *Culicidae* genome database (Taxid: 7157) using Kraken2. Host-depleted reads were profiled against the Kraken2 (v2.1.3) PlusPFp database (by 9 October 2023). Species-level abundances were then corrected for potential false positives and re-estimated using Bracken (v2.9), with these Bracken-corrected counts used for all downstream statistical analyses. The species level was adopted as the minimal quantifiable taxonomic unit. *De novo* assembly of reads was performed using MEGAHIT (v1.2.9), and contigs were polished with Pilon (v1.24). Contigs were annotated via BLASTN/BLASTX (v2.14.1+) against the NCBI nt/nr database (E-value < 1e^−5^). For viral quantification, which is prone to Kraken inaccuracies, an assembly-based approach was implemented. Viral reads were aligned to assembled viral contigs using Bowtie2 (v2.5.1; --very-sensitive-local mode). Only contigs >500 bp with >30% read coverage were retained. Viral abundance was calculated using reads per kilobase million with BBMap (v39.01) to normalize for contig length and sequencing depth, effectively replacing Kraken-derived viral counts. Visualizations included interactive radial plots (KronaTools, v2.8), circular cladograms (GraPhlAn, v1.1.3), and relative abundance charts (ggplot2, v3.4.2 in R).

### Identification and visualization of core microbial taxa

Core microbial taxa were defined using a combined prevalence-abundance threshold, implemented via the core function in the microbiome R package (v1.21.0). Taxa were designated as core members if they exceeded a relative abundance of 0.1% in more than 10% of all samples. To visualize prevalence and abundance patterns across taxa, we employed plot_taxa_prevalence() at the phylum level.

### Alpha diversity estimation and rarefaction

Alpha diversity was assessed using the Shannon index (phyloseq R package). Rarefaction curves were computed using mp_cal_rarecurve() from MicrobiotaProcess to confirm sequencing saturation. The effect of ecological covariates (location, time, host genus, sex) on diversity was tested using one-way ANOVA and stepwise regression [stepAIC() in MASS].

### Beta diversity, community structure, and variance decomposition

To explore patterns of microbial community composition across mosquito populations, Bray-Curtis dissimilarity matrices were calculated from relative abundance data. Beta diversity was quantified using Bray-Curtis dissimilarity. Community clustering was visualized via PCoA [ordinate() in phyloseq]. The statistical influence of covariates was determined using PERMANOVA [adonis2() in vegan; 999 permutations] and constrained by db-RDA [capscale()]. Variance partitioning was conducted with varpart() to disentangle the unique and shared contributions of spatial, temporal, and host-related factors, with fractions visualized using UpSetR.

### Microbial co-occurrence network inference and structural analysis

Microbial networks were inferred using SparCC correlations (ggClusterNet R package, v1.1.3), retaining edges with |*r*| >0.6 and *P* <0.05 (10 bootstrap iterations). Node centrality (degree, betweenness, closeness) was computed with igraph. Keystone taxa were identified using the Zi-Pi framework, where Zi (within-module degree) and Pi (participation coefficient) assigned nodes to ecological roles: network hubs (Zi > 2.5, Pi > 0.62), module hubs (Zi > 2.5, Pi < 0.62), connectors (Zi < 2.5, Pi > 0.62), and peripherals (Zi < 2.5, Pi < 0.62). Network stability was assessed by simulating targeted node removal using natural.con.microp().

### Phylogenetic analysis

Viral contigs were screened for key domains (RdRp via RdRpscan HMM database; DNA replicases/NS1 via conserved HMM profiles). For phylogenetic analysis, sequences were clustered at 95% amino acid identity using MMseqs, and the longest contig from each cluster was selected as the representative sequence. Multiple sequence alignments were performed with MAFFT (v7.490) and trimmed with TrimAl (v1.4.rev15). Phylogenetic trees were inferred using FastTree (v2.1.11) and visualized with ggtree. Critically, reassortment analyses were conducted using nucleotide-based phylogenies at the intra-species level to accurately detect segment-specific evolutionary events. Viral host taxonomy was annotated using the Virus-Host DB and integrated into tree visualizations.

## Data Availability

The raw sequencing reads and viral contigs are publicly available under GSA accession CRA027708 and GenBase accessions C_AA111730.1–C_AA112024.1, respectively, at the National Genomics Data Center (NGDC).

## SUPPLEMENTAL MATERIAL

Fig. S1 msystems.01427-25-s0001.docx Alpha diversity of bacterial and viral communities in Kenyan mosquitoes.

Fig. S2 msystems.01427-25-s0002.docx Phylogenetic analysis of *Culex* and *Aedes* mosquitoes using mitochondrial COI sequences.

Table S1 msystems.01427-25-s0003.xlsx Detailed metadata and pooling strategy for metatranscriptomic samples.

Table S2 msystems.01427-25-s0004.xlsx Comprehensive abundance table of all microbial taxa identified in Kenyan mosquitoes.

ASM does not own the copyrights to Supplemental Material that may be linked to, or accessed through, an article. The authors have granted ASM a non-exclusive, world-wide license to publish the Supplemental Material files. Please contact the corresponding author directly for reuse.
