# Supplementary material for: Host and geography shape microbial communities in Kenyan mosquitoes: insights from metatranscriptomics
Source: mSystems. 2026 Jan 20;11(2):e01427-25. doi: 10.1128/msystems.01427-25 (PMC12911392; doi:10.1128/msystems.01427-25)
Supplement: Fig. S1 — Alpha diversity of bacterial and viral communities in Kenyan mosquitoes. [file msystems.01427-25-s0001.docx]

**
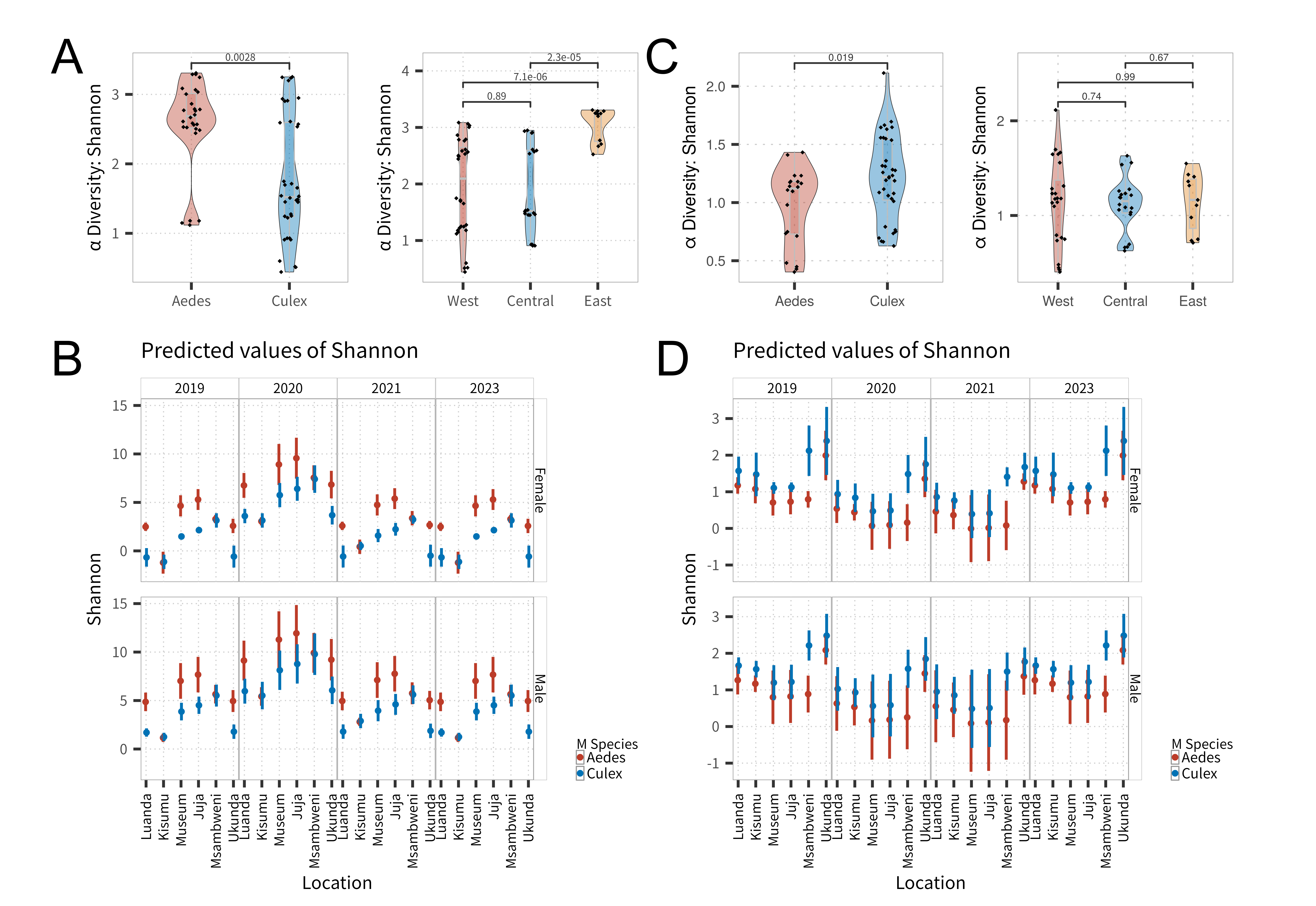
**

**Fig. S1 Alpha diversity of bacterial and viral communities in Kenyan mosquitoes.** (A) Boxplots and jittered dot plots comparing Shannon diversity of bacterial communities across mosquito genus (Aedes vs. Culex, left) and across geographic regions (right). Statistical significance is indicated by Wilcoxon rank-sum test (p values). (B) Predicted Shannon diversity values for bacterial communities across mosquito genus within each geographic region and sex, based on stepwise AIC modeling. (C) Boxplots and dot plots for viral Shannon diversity across mosquito genus (left) and regions (right), with Wilcoxon test significance. (D) Predicted Shannon diversity values for viral communities across mosquito genus within each region and sex, as estimated by stepwise AIC modeling.
