# Supplementary material for: Host and geography shape microbial communities in Kenyan mosquitoes: insights from metatranscriptomics
Source: mSystems. 2026 Jan 20;11(2):e01427-25. doi: 10.1128/msystems.01427-25 (PMC12911392; doi:10.1128/msystems.01427-25)
Supplement: Fig. S2 — Phylogenetic analysis of Culex and Aedes mosquitoes using mitochondrial COI sequences. [file msystems.01427-25-s0002.docx]

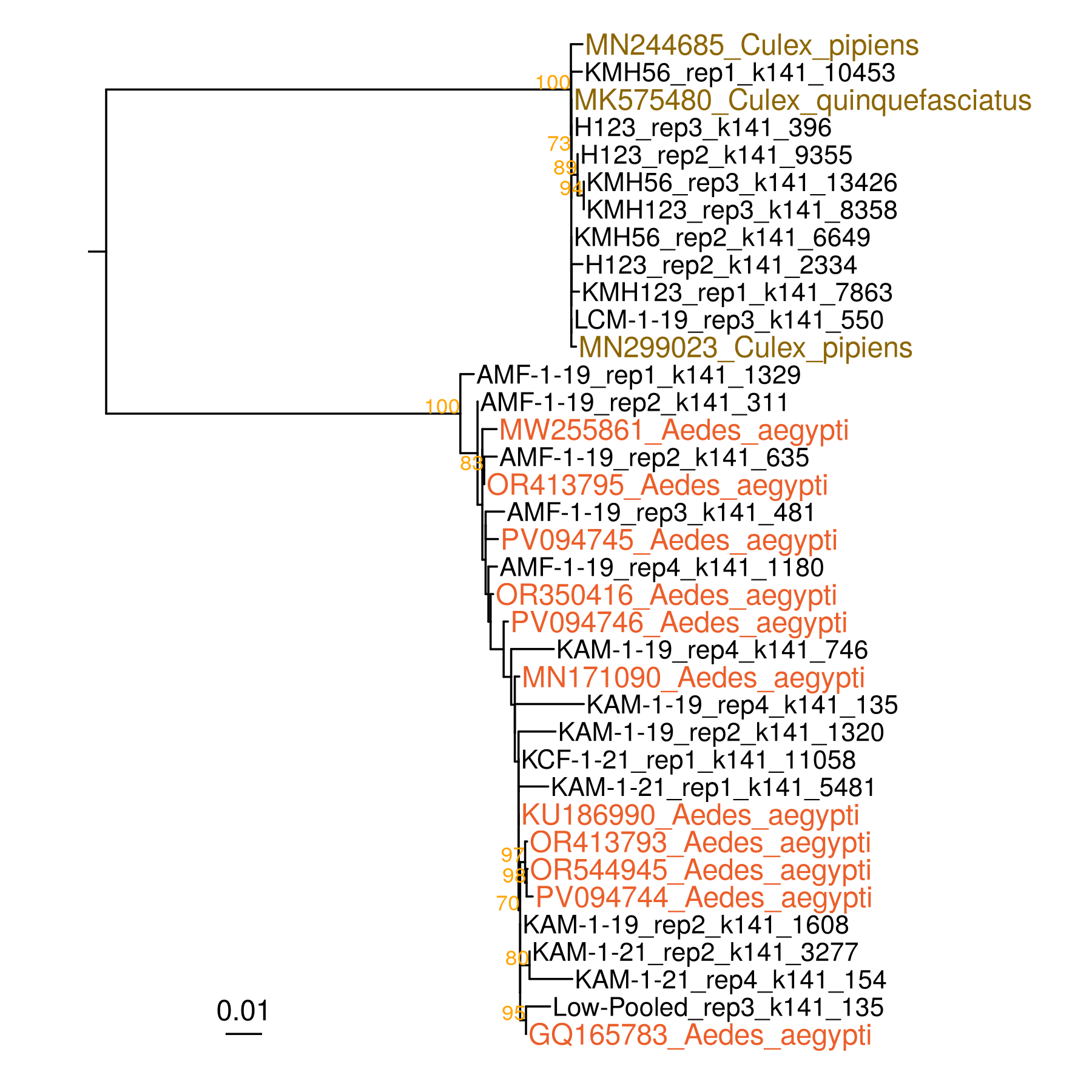


**Fig. S2 Phylogenetic analysis of *Culex* and *Aedes* mosquitoes using mitochondrial COI sequences derived from assembled high-throughput contigs and NCBI references**. The maximum-likelihood (ML) tree was constructed using IQ-TREE following MAFFT alignment and TrimAl trimming, showing two distinct clades labeled in light brown (Culex) and orange (Aedes), with node support indicated by UFBoot values greater than 70.
